# Orbital Complications of Rhinosinusitis in Adults: A 10-Year Retrospective Cohort Study

**DOI:** 10.7759/cureus.86868

**Published:** 2025-06-27

**Authors:** José Alberto Fernandes, António Andrade, Pedro Valente, Ricardo Vaz

**Affiliations:** 1 Department of Otorhinolaryngology, Unidade Local de Saúde de São João, Porto, PRT; 2 Unit of Otorhinolaryngology, Department of Surgery and Physiology, Faculty of Medicine, University of Porto, Porto, PRT; 3 Department of Otorhinolaryngology, Unidade Local de Saúde de Trás-os-Montes e Alto Douro, Vila Real, PRT; 4 Unit of Anatomy, Department of Biomedicine, Faculty of Medicine, University of Porto, Porto, PRT; 5 NeuroGen Research Group, Center for Health Technology and Services Research (CINTESIS), Porto, PRT; 6 CINTESIS@RISE, Faculty of Medicine, University of Porto, Porto, PRT

**Keywords:** adult infections, chandler classification, endoscopic sinus surgery (fess), orbital complications, rhinosinusitis

## Abstract

Background

Orbital complications of rhinosinusitis are rare in adults but may result in serious outcomes such as vision loss or intracranial involvement. While more frequently reported in children, adult cases tend to present with greater severity. Early recognition and appropriate management are essential. This study aims to describe the clinical characteristics, diagnostic methods, treatment strategies, and outcomes of adult patients with orbital complications secondary to acute or chronic rhinosinusitis.

Methodology

A retrospective cohort study was conducted among 28 adults admitted to a tertiary hospital between 2012 and 2022 with CT-confirmed orbital complications of rhinosinusitis. Data included demographics, comorbidities, Chandler stage, prior sinus surgery, treatments, and outcomes.

Results

Most patients were male (60.7%), with a mean age of 44.3 years. Acute rhinosinusitis (67.9%) was more common than chronic (32.1%). Patients with advanced Chandler stages III-IV were significantly older (mean age = 62 vs. 35 years; p = 0.002). Comorbidities and previous outpatient antibiotic use showed no association with severity. Among chronic rhinosinusitis cases, 55.5% had a history of endoscopic sinus surgery; however, this was not associated with worse outcomes. Over half of the patients (53.4%) were treated successfully with intravenous antibiotics alone, while 46.4% required surgical intervention. No patients developed cavernous sinus thrombosis, and all achieved full recovery without long-term sequelae.

Conclusions

Orbital complications of rhinosinusitis in adults are uncommon but clinically significant. Age is a key predictor of severity. Early-stage cases can often be managed conservatively, while advanced cases require surgery. Early imaging and multidisciplinary care are critical for optimal outcomes.

## Introduction

Although well documented in pediatric populations, orbital complications secondary to rhinosinusitis are relatively uncommon in adults. This lower incidence contrasts with pediatric cases, where orbital complications are more frequently reported, primarily due to anatomical and immunological factors that favor the spread of infection from the paranasal sinuses to the orbit. In adults, however, when orbital involvement does occur, it tends to present with greater clinical severity and a higher likelihood of requiring surgical intervention [1,2].

Despite their lower incidence in adults, orbital complications such as preseptal and orbital cellulitis, subperiosteal, orbital abscesses, and even intracranial extension, including meningitis or cavernous sinus thrombosis, can result in significant morbidity and potential vision loss if not promptly diagnosed and adequately managed [3,4]. The anatomical proximity between the orbit and paranasal sinuses, coupled with thin bony barriers and a valveless venous plexus, facilitates the spread of infection, making early identification and intervention critical.

The diagnosis of orbital involvement typically relies on a combination of clinical evaluation and radiological imaging, most notably CT, which provides rapid and accurate assessment of sinus pathology and orbital extension. Management strategies differ according to the severity of the condition, often guided by the Chandler classification system, ranging from conservative antibiotic therapy to urgent surgical drainage in advanced cases [5].

While numerous studies in the current literature focus on pediatric populations, data on adult presentations remain limited, and evidence-based guidelines for this demographic are less well established. This underscores the need for further investigation into the clinical presentation, risk factors, diagnostic pathways, and treatment outcomes specific to adults.

The present study aims to characterize the demographic and clinical profiles, diagnostic approaches, therapeutic interventions, and outcomes of adult patients presenting with orbital complications of acute and chronic rhinosinusitis at a tertiary care center over a 10-year period.

## Materials and methods

A retrospective cohort study was conducted among all adult patients (>18 years) admitted to the Department of Otorhinolaryngology at a tertiary hospital between January 2012 and December 2022 with a confirmed diagnosis of rhinosinusitis complicated by orbital involvement. Diagnostic confirmation was established through clinical assessment and imaging studies, primarily CT, with orbital complications classified according to the Chandler classification.

Rhinosinusitis was classified according to the duration and clinical characteristics of symptoms, based on established international guidelines, including the European Position Paper on Rhinosinusitis and Nasal Polyps 2020 (EPOS 2020) and the International Consensus Statement on Allergy and Rhinology: Rhinosinusitis 2021 (ICAR-RS 2021). Acute rhinosinusitis (ARS) was defined as an inflammatory condition of the nasal and paranasal mucosa lasting fewer than 12 weeks, typically resolving completely, and most commonly of viral etiology, although some cases progressed into post-viral or bacterial forms. Chronic rhinosinusitis (CRS) was characterized by persistent sinonasal symptoms lasting 12 weeks or more without complete resolution, and was further subclassified into CRS with nasal polyps (CRSwNP) and CRS without nasal polyps (CRSsNP).

Data collected included demographic characteristics, comorbidities, history of prior sinus surgery, presenting symptoms, radiologic findings, Chandler classification, antibiotic treatment, surgical interventions, length of hospital stay, and clinical outcomes.

All data were analyzed using SPSS Statistics version 26.0 (IBM Corp., Armonk, NY, USA). Descriptive statistics were used to summarize demographic and clinical variables, with means and standard deviations calculated for continuous variables and frequencies and percentages for categorical variables. The Shapiro-Wilk test was employed to assess the normality of continuous variables. For group comparisons, Student’s t-test was applied for normally distributed continuous variables, while the Mann-Whitney U test was used for non-normally distributed data. Associations between categorical variables were assessed using the chi-square test or Fisher’s exact test, as appropriate. A p-value <0.05 was considered statistically significant.

This study was approved by the Ethics Committee of Unidade de Saúde de São João, Porto, Portugal (approval number: CE 185-2025). All procedures involving human participants complied with the ethical standards of the institutional or national research committee, as well as the 1964 Declaration of Helsinki and its subsequent amendments. Owing to the retrospective nature of the study, the requirement for informed consent was waived by the Ethics Committee.

## Results

A total of 28 adult patients diagnosed with orbital complications secondary to rhinosinusitis were included in the cohort. Of these, 60.7% (n = 17) were male and 39.3% (n = 11) female. The mean age was 43.6 years (SD ± 18.45) in males and 51.3 years (SD ± 26.46) in females.

**Figure 1:**
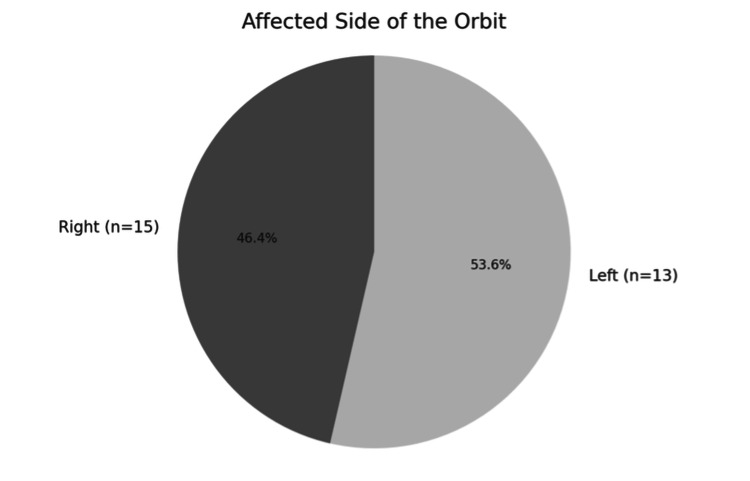
Laterality distribution of orbital involvement.

Laterality analysis revealed that 53.6% of cases (n = 15) involved the left orbit, while 46.4% (n = 13) affected the right orbit, with no significant lateral predominance (Figure 1).

Comorbidities were present in 21% (n = 6) of patients, including diabetes mellitus, asthma, and states of immunosuppression. However, the presence of these underlying conditions was not significantly associated with increased severity of orbital involvement, as assessed by the Chandler classification (p = 0.275).

Clinically, all patients exhibited periorbital edema and erythema (100%), while proptosis was observed in 17% (n = 4), and ophthalmoplegia in 4.5% (n = 1). Additionally, all patients also reported sinonasal symptoms consistent with acute rhinosinusitis, including nasal obstruction, purulent rhinorrhea, headache, and cough (Table 1).

**Table 1:** Clinical presentation. ARS: acute rhinosinusitis; CRS: chronic rhinosinusitis

Symptoms% (n)	ARS, % (n)	CRS, % (n)	Total, % (n)
Swelling and erythema	61% (n = 17)	39% (n = 11)	100% (n = 28)
Proptosis	12% (n = 3)	5% (n = 1)	17% (n = 4)
Ophthalmoplegia	4.5% (n = 1)	-	4.5% (n = 1)

Following the criteria of the Chandler classification, stage II (orbital cellulitis) was the most prevalent, observed in 42.9% of cases (n = 12), followed by stage I (preseptal cellulitis) in 28.6% (n = 8), stage III (subperiosteal abscess) in 17.9% (n = 5), and stage IV (orbital abscess) in 10.7% (n = 3). No instances of stage V (cavernous sinus thrombosis) were documented (Table 2).

**Table 2:** Clinical presentation based on the Chandler classification.

Chandler classification, % (n)	% (n)
I (preseptal cellulitis)	28.6% (n = 8)
II (orbital cellulitis)	42.9% (n = 12)
III (subperiosteal abscess)	17.9% (n = 5)
IV (orbital abscess)	10.7% (n = 3)
V (cavernous sinus thrombosis)	-

ARS accounted for the majority of cases (67%), whereas CRS was identified in 33.1% of patients. Individuals with ARS were significantly younger than those with CRS (mean age = 32.0 vs. 51.5 years; p = 0.032). Furthermore, a significant association was also found between patient age and the severity of orbital complications. The mean age for Chandler stages I-II was 35 years, compared to 62 years among those with stage III-IV involvement (p = 0.002). These values are presented in Table 3.

**Table 3:** Distribution of clinical presentation between the ARS and CRS groups. ARS: acute rhinosinusitis; CRS: chronic rhinosinusitis

Stage	ARS, % (n)	CRS, % (n)	Total, % (n)
I (preseptal cellulitis)	25% (n = 7)	3.6% (n = 1)	28.6% (n = 8)
II (orbital cellulitis)	32% (n = 9)	10.9% (n = 3)	42.9% (n = 12)
III (subperiosteal abscess)	10% (n = 3)	7.9% (n = 2)	17.9% (n = 5)
IV (orbital abscess)	-	10.7% (n = 3)	10.7% (n = 3)
V (cavernous sinus thrombosis)	-	-	-

Pre-admission empirical antibiotic therapy was administered in 43% of cases (n = 12), with amoxicillin-clavulanate representing the most commonly prescribed regimen (39.3%), followed by ceftriaxone (3.6%). Nevertheless, prior antibiotic treatment did not demonstrate a statistically significant protective effect against progression to severe orbital disease (p = 0.401).

Among patients diagnosed with CRS, 55.5% (n = 5) had a history of endoscopic sinus surgery. Postoperative imaging frequently revealed bony dehiscence, particularly involving the lamina papyracea and frontal sinus walls. Despite these anatomical changes, no statistically significant correlation was observed between previous surgical intervention and the severity of orbital complications (p = 0.540).

Medical management with intravenous antibiotic therapy resulted in complete clinical resolution in 53.4% of patients (n = 15). Among those successfully managed conservatively, 53.3% (n = 8) received combination therapy with ceftriaxone and clindamycin, 40% (n = 6) received ceftriaxone monotherapy, and 6.7% (n = 1) were treated with ceftriaxone in combination with vancomycin.

Conversely, 46.4% of patients (n = 13) exhibited either clinical worsening or lack of improvement within 24-48 hours of antibiotic initiation and consequently required surgical intervention. Subperiosteal abscess drainage via endoscopic sinus surgery (ESS) was performed in 17.8% (n = 5), while 28.6% (n = 8) underwent functional ESS for local infection control. Orbital abscesses, identified in 10.7% of patients (n = 3), were surgically managed by the Department of Ophthalmology.

The mean length of hospital stay was approximately 10.6 days. Notably, all patients experienced full clinical recovery without complications or long-term sequelae during the follow-up period. No cases of cavernous sinus thrombosis or intracranial extension were reported.

## Discussion

The findings of this study are consistent with previous reports, reinforcing prior evidence that older patients with rhinosinusitis are at an increased risk of developing severe orbital complications. The statistically significant correlation between advanced age and higher Chandler stages (III-IV) aligns with prior findings suggesting that immunosenescence and age-related comorbidities contribute to more aggressive disease progression and delayed therapeutic responses [6,7]. This trend may also explain the higher surgical intervention rate observed in adult populations, despite the predominance of pediatric data in the literature [2,4]. Furthermore, pediatric studies have reported earlier diagnosis and better antibiotic response, which may contribute to lower complication rates in younger patients [8].

While CRS and prior endoscopic sinus surgery were relatively frequent among patients, neither was associated with worse clinical outcomes in this cohort. This observation is consistent with El Mograbi et al. (2019), who found no significant relationship between previous sinus surgery and poor prognosis in adult orbital complications [1]. Although anatomical alterations such as bony dehiscence could theoretically increase the risk of orbital involvement, our findings suggest that such factors alone are insufficient determinants of severity, particularly in the absence of diagnostic delay or immunodeficiency [9,10].

Empirical outpatient antibiotic therapy, most often involving amoxicillin-clavulanate, did not significantly reduce disease severity at admission. This may reflect the limited efficacy of empirical regimens against polymicrobial or resistant organisms commonly implicated in these infections [3]. Moreover, as highlighted in previous studies, delays in diagnosis and imaging remain critical contributors to progression and complications [11,12]. These findings underscore the importance of prompt radiological assessment and early referral to specialized care.

Our results further validate the utility of the Chandler classification in guiding clinical decision-making. Consistent with Chandler’s original framework, patients in stages I-II were effectively managed with conservative therapy, whereas those in stages III-IV frequently required surgical intervention [5]. The proportion of surgical cases in our cohort (46.4%) aligns with rates reported in similar adult populations [3,13].

Notably, no cases of cavernous sinus thrombosis or permanent visual impairment occurred, which may be attributable to early multidisciplinary intervention and coordinated management involving otolaryngology, ophthalmology, and infectious disease teams. Integrated care pathways have been shown to improve outcomes in orbital infections [10].

This study is limited by its retrospective design and modest sample size, potentially reducing statistical power and limiting the generalizability of our findings. Additionally, the absence of microbiological culture data from surgical specimens limited the ability to analyze pathogen-specific patterns that could inform antimicrobial therapy. Future prospective studies with larger patient cohorts and culture-based microbiological evidence are warranted to further delineate risk factors and optimize treatment algorithms for adult orbital complications of rhinosinusitis.

## Conclusions

Although rare, orbital complications of rhinosinusitis in adults remain clinically challenging and may lead to severe outcomes. Among known risk factors, age remains a significant predictor of complication severity. While conservative management is appropriate for early-stage disease, surgical intervention is essential in refractory or advanced cases. Early diagnosis, radiologic assessment, and a multidisciplinary approach are key to favorable outcomes. Further prospective studies with larger cohorts are needed to better elucidate risk factors and refine management strategies.
